# An Integrative Review of Potential Diagnostic Biomarkers for Complex Regional Pain Syndrome

**DOI:** 10.3390/jcm14113751

**Published:** 2025-05-27

**Authors:** Revelino Lopes, André Santos, Teresa Gomes, Júlia Ribeiro, Ivone Rodrigues, Bruno Paiva, Isa Nzwalo, Deise Catamo, Jamal Baco, Helena Buque, Marta Botelho, Sandra Pais, Hipólito Nzwalo

**Affiliations:** 1 Faculty of Medicine and Biomedical Sciences, University of Algarve, 8005-139 Faro, Portugal; isanzwalo@gmail.com (I.N.); deisehawavaz@gmail.com (D.C.); jamalbaco8@gmail.com (J.B.); helenabuque@gmail.com (H.B.); marta.botelho@abcmedicalg.pt (M.B.); 2 Faro Unit Hospital, Local Health Unit of Algarve, 8000-386 Faro, Portugal; ajfreitassantos@gmail.com (A.S.); tetercg@gmail.com (T.G.); julia.machado.ribeiro.md@gmail.com (J.R.); ivone.rodrigues@ulsalg.min-saude.pt (I.R.); brunopaiva.mfr@gmail.com (B.P.); 3 Aging and Cerebrovascular Research Group, Algarve Biomedical Research Institute, 8005-139 Faro, Portugal; 4 Algarve Biomedical Center, 8005-139 Faro, Portugal; 5 Comprehensive Health Research Centre-CHRC, University of Évora, 7004-516 Évora, Portugal; spais@uevora.pt

**Keywords:** complex regional pain syndrome, biomarkers, cytokines, neuropeptides, inflammation, immune response

## Abstract

**Background:** Complex regional pain syndrome (CRPS) is a rare, chronic, painful, neurological, debilitating disorder. Despite the substantial impact on quality of life, diagnosis remains challenging due to its complex pathophysiology and subjective clinical criteria. This integrative review aims to synthesize current research on potential diagnostic biomarkers for CRPS. **Methods:** A systematic search was conducted using the PubMed and Scopus databases to identify relevant studies published until January 2025. Inclusion criteria focused on adult CRPS patients, with studies examining diagnostic or predictive biomarkers. **Results:** Key findings highlight the role of inflammatory and immune-related biomarkers, such as elevated levels of cytokines (IL-6, TNF-α), immune cell infiltration, and specific autoantibodies. Neuropeptides, including substance P and calcitonin gene-related peptide, were associated with pain sensitization in acute phases, though their levels normalized in chronic stages. Additionally, genetic and epigenetic markers, brain imaging, and neurophysiological alterations provided insights into CRPS pathogenesis, emphasizing the dynamic nature of these biomarkers across disease stages. **Conclusions:** This review underscores the need for further research to integrate these biomarkers into diagnostic frameworks, which could enhance early diagnosis and treatment strategies for CRPS.

## 1. Introduction

Complex regional pain syndrome (CRPS), formerly known as algodystrophy, causalgia (type 2 CRPS), reflex sympathetic dystrophy (type 1 CRPS), and Sudeck’s atrophy, is a chronic neurologic condition that is frequently precipitated by trauma or elective surgery to the upper and lower limbs. CRPS causes significant morbidity and loss of quality of life [1]. The pooled global prevalence at 12 and 24 months is 3.04% and 6.46%, respectively, in at-risk populations, challenging the current idea that CRPS is a rare condition [2].

The Budapest criteria for CRPS considers five categories of symptoms, pain disproportionate to any inciting event, sensory (allodynia, hyperalgesia), vasomotor changes (temperature asymmetry, skin color changes), sudomotor/edema changes (edema, sweating changes), and motor/trophic changes (decreased range of motion, muscle atrophy). A person with at least one symptom in three of the four categories is diagnosed with CRPS [3]. Management of CRPS is multimodal, often including a combination of free-radical scavengers, steroids and nonsteroidal anti-inflammatory medications, anti-depressants, gabapentinoids, and physiotherapy [4]. These interventions are more effective in the early stages of the disease [4,5].

Other interventions, such as intravenous regional blocks, spinal cord stimulation, dorsal root ganglion stimulation, and regional sympathetic nerve blocks, including stellate ganglion block, can be considered in severe CRPS [6].

For unknown reasons, the clinical course of CRPS varies significantly with spontaneous resolution in one pole and aggressive refractory disease at the opposite pole, despite adequate management [5]. The uncertainty about clinical evolution reflects the complexity of the underlying pathophysiology that remains to be clarified. Nevertheless, there is consensus that CRPS results from aberrant peripheral and central responses to an injury which may involve multifactorial contribution of genetic predisposition, inflammation, immunological dysfunction, and brain plasticity adaptations [7,8,9].

Despite growing interest in the pathophysiological mechanisms of CRPS, there remains a significant knowledge gap regarding reliable diagnostic biomarkers for the condition. Current evidence is fragmented, with studies often limited by small sample sizes, heterogeneous methodologies, and a lack of replication. While several potential biomarkers, including inflammatory cytokines, neuropeptides, genetic markers, and imaging findings, have been proposed, none have demonstrated consistent diagnostic accuracy or clinical utility.

Previous reviews were focused on brain imaging biomarkers [10] or other specific biomarkers such as inflammation [11,12] and some of the reviews have been outdated for more than a decade [12,13]. The integration of different diagnostic biomarkers can reduce subjectiveness and anticipate diagnosis and treatment. For these reasons, we sought to systematically review the available data on the potential diagnosis biomarkers of CRPS.

## 2. Materials and Methods

Methods Search Strategy: Pubmed and Scopus databases were used to search for relevant publications from interception to 30 January 2025 using the following terms Algoneurodystrophy, “Sudeck syndrome”, “causalgia” and “Complex Regional Pain Syndrome”, “reflex sympathetic dystrophy”, “reflex neurovascular dystrophy”. We have complemented this search by examining reference lists of the most relevant studies and the Open Grey database (http://www.opengrey.eu/ accessed on 3 March 2025). Based on the PICO framework, with individuals diagnosed with CRPS as the Population; potential diagnostic biomarkers as Intervention/Exposure; healthy individuals, the contralateral normal limb, and other pain conditions as the Comparison; and the direction of biomarker association as the Outcome (increased, decreased, or no association), we extracted information on the total number of cases, the specific biomarkers investigated, and the qualitative interpretation of their associations (i.e., whether they were increased, decreased, or showed no association with CRPS.

Study selection: We included prospective and retrospective studies published up to 30 January 2025, containing information on one of the following domains under analysis: frequency and diagnostic or predictive associated factors. Only studies focusing on CRPS adult populations (≥18 years) and written in English were taken into consideration. Conference or seminar abstracts and/or studies with unclear inclusion criteria or convenience sampling, including very selective groups or mixed populations with non-CRPS patients, were excluded from the selection. Two authors independently screened abstracts obtained from the database search. Discrepancies were evaluated by a third investigator and resolved by the main investigator (RL). The full texts of potentially relevant articles were retrieved for further assessment. The Preferred Reporting Items for Systematic Reviews and Meta-Analyses (PRISMA) checklist for systematic reviews was used to guide data extraction and reporting.

### 2.1. Risk of Bias Assessment

Two investigators independently evaluated the risk of bias in the studies using the Newcastle–Ottawa Scale (NOS). The NOS includes three categories: selection of study groups, comparability of groups, and measurement of exposure, with a maximum score of 9. Scores of 0–4 are considered low quality (high risk of bias), 5–6 as moderate quality (moderate risk of bias), and 7–9 as high quality (low risk of bias) [14].

### 2.2. Data Extraction and Synthesis

Data were analyzed qualitatively. A priori, we divided biomarkers into inflammation and immune-related potential biomarkers (cytokines, cells, autoantibodies, neuropeptides, and others); genetic and epigenetic; brain imaging, and functional neurophysiological biomarkers. When appropriate data were described in terms of frequency, means, and median. No meta-analysis was anticipated due to the expected marked heterogeneity and methodological variability of the studies. The study protocol was registered at Prospero (https://www.crd.york.ac.uk/PROSPERO/ accessed on 3 March 2025) with the number CRD4389417.

## 3. Results

**Figure 1:**
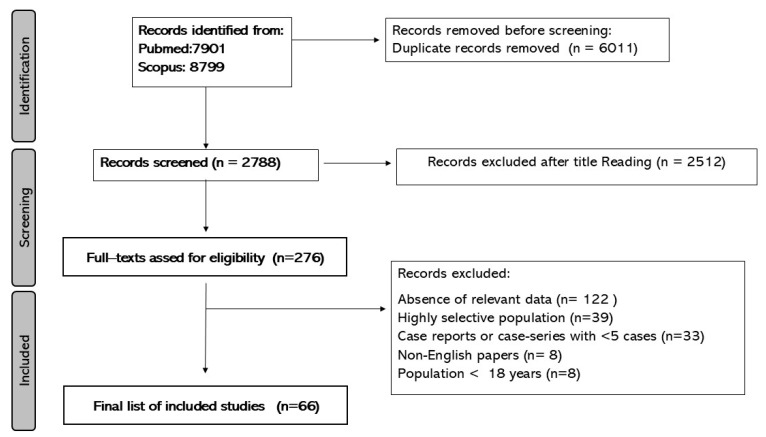
PRISMA-Pflow chart of the study inclusion process.

We identified a total of 7901 (Pubmed) and 8799 (Scopus) publications using the predefined searching criteria. Preferred Reporting Items for Systematic Reviews and Meta-Analyses (PRISMA) flowchart diagram (Figure 1) resumes the selection and inclusion process. A total of 66 [9,11,15,16,17,18,19,20,21,22,23,24,25,26,27,28,29,30,31,32,33,34,35,36,37,38,39,40,41,42,43,44,45,46,47,48,49,50,51,52,53,54,55,56,57,58,59,60,61,62,63,64,65,66,67,68,69,70,71,72,73,74,75,76,77,78] studies, 2691 patients with CRPS were included in the systematic review.

**Figure 2:**
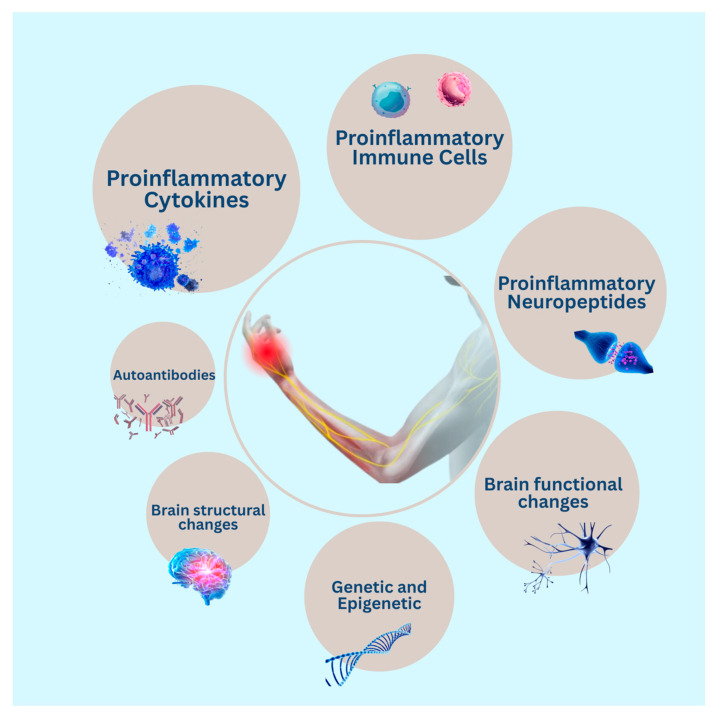
List of potential diagnostic biomarkers for complex regional pain syndrome identified in the systematic review.

The studies addressed different types of potential inflammation, immune, genetic, epigenetic, brain imaging, and functional neurophysiological biomarkers associated with complex regional pain syndrome (Figure 2).

The list of inflammation and immune-related potential biomarkers found in the literature is depicted in Table 1.

### 3.1. Immune System Cells

Most studies showed that the systemic distribution of immune system cells, namely B and T lymphocytes, natural killers, and monocytes/macrophages is similar between CRPS patients and controls [46,60,61,64,65,72,77]. However, when comparing specific or subsets of immune cell populations, an increased number of proinflammatory CD14+ CD16+ monocytes [61] and a reduction in cytotoxic CD8+ lymphocytes and IL-2-producing T cells [46] was demonstrated in CRPS patients. In studies based on cutaneous or subcutaneous tissue samples from the affected limb, an increased number of leukocytes [69] and mast cells [23] was demonstrated in patients with CRPS.

In addition, the number of central memory CD8+, and CD4+ T lymphocytes was found to be reduced in patients with CRPS in comparison to controls [63].

### 3.2. Autoantibodies

The prevalence of autoantibodies against the myenteric plexus [24], neurons [30], autonomic nervous system antigens [49,50], and tumor suppressor P29ING4 [19] was found to be elevated in CRPS patients. In addition, in a single study, the frequency of anti-nuclear antibodies was higher in CRPS patients in comparison to controls [30]. In none of these studies was the pathogenicity proven.

### 3.3. Cytokines

Table 1 shows that 14 studies evaluated the levels of different cytokines between CRPS patients and controls, with IL-6 and TNF-α being the most frequently studied. The systemic levels of IL-6 [57,64,65,72] and TNF-α [57,64,72] were comparable to controls. On the contrary, the systemic levels of IL-8 [65], and IL-2 [72] were higher in patients with CRPS. The systemic levels of IL-10 and transforming growth factor beta 1 [72], granulocyte-macrophage colony-stimulating factor, and IL-37 were decreased in CRPS [62] in comparison to controls. The systemic levels of IL-4, IL-8, IL-10, IL-11, and IL-12 were also found to be similar between cases and controls [57,64].

In all studies evaluating the behavior of cytokines in skin blisters, the levels of pro-inflammatory cytokines TNF-α and IL-6 [23,30,35,40,41,56,72,76] were found to be elevated in the acute and intermediate stages, but not in the chronic phase [56,76]. The local levels of eotaxin, an eosinophilic chemokine, were found to be diminished in a single study [35]. No local differences for IL-1b, IL-1b [40], IFNγ, IL-2, IL-2R, IL-4, IL-5, and IL-10 [35].

In the cerebrospinal fluid, the levels of IL-1, IL-2, IL-6, IL-10, and monocyte chemotactic protein-1 were elevated [16,17], whereas the level of TNF-α was normal [17] in patients with CRPS.

### 3.4. Other Immune-Related Proteins

The level of different proteins associated with specific immune cell activation, such as the soluble IL receptor and selectins for activated T cells [11,64,65], and of local [23,41], but not the systemic [73] tryptase for mast cells was altered in CRPS patients. The C reactive protein level was normal in all studies [64,65,72,77] (Table 1).

### 3.5. Neuropeptides and Neurogenic Inflammation

In 4 studies (Table 1), the local and systemic levels of neuropeptides namely CGRP, substance P, neuropeptide Y, neurokinin, and bradykinin side were higher in CRPS patients in comparison to controls or the contra-lateral healthy side [13,22,25,64]. The behavior of neuropeptides was found to change over time, with studies showing normalization of CGRP associated with improvement of local inflammation [22], or absence of differences between CRPS and controls or even lower levels in patients with chronic CRPS [64,65]. For substance P, the shift was also demonstrated with increased levels in acute [64,65] and normal levels in chronic patients [64].

The level of different proteins associated with specific immune cell activation such as the soluble IL receptor and selectins for activated T cells [11,64,65], and of local [23,41], but not the systemic [73] tryptase for mast cells was altered in CRPS patients. The C reactive protein level was normal in all studies [64,65,72,77]. The levels of anandamide which is synthesized by nucleated blood cells are higher in long-lasting CRPS suggesting an effort of the endogenous cannabinoid system to modulate neuropathic pain and pain memory [47].

Table 2 depicts the genetic, epigenetic, brain imaging, and functional neurophysiological biomarkers associated with complex regional pain syndrome.

### 3.6. Genetic and Epigenetics

In one study, five top hub genes: MMP9, PTGS2, CXCL8, OSM, and TLN1 were identified to be correlated with the development of CRPS [9].

Specific microRNA, hsa-miR-532-3p, were found in patients with increased vascular endothelial growth factor [57] in patients with CRPS. In another study, no correlation between transforming growth factor-b1mRNA and CRPS was found [72].

### 3.7. Brain Structural and Functional Alterations

Volume reduction in grey matter in the somatosensory cortex, limbic system, prefrontal cortex, and pain-related areas in brain magnetic resonance was demonstrated in CRPS patients [20,31,33,54,68] (Table 2). A single study demonstrated an increased volume of choroid plexus in patients with CRPS [78]. The density of gray matter in the dorsomedial prefrontal cortex was found to be increased in a single study [59].

Disruption of interactions between specific central and metabolic metabolites in the thalamus was reported in one study [44] (Table 2). A low perfusion in the somatosensory cortex and limbic system (early phase) and a high perfusion in the somatosensory cortex, and limbic system (late phase) were shown in patients with CRPS [68] and high activity in somatosensory cortex and low activity in specific motor areas was also described in CRPS patients [67] (Table 2). Table 2 shows that in 11 studies, functional brain alterations such as sustained somatotopic alteration of the somatosensory cortex [38,39]; high localized activation in the primary somatosensory cortex [27,55,70,71]; increased functional connectivity in the somatosensory subnetworks and low functional connectivity in the prefronto-parieto-cingulo-thalamic subnetworks [18,37]; high Basal ganglia infra-slow oscillations and resting connectivity [53]; increased thalami functional connectivity [28]; diminished activation of subthalamic nucleus, nucleus accumbens, and putamen [48].

### 3.8. Other Biomarkers

The presence of high NMDA excitatory amino acids (glutamate, glutamine, glycine, taurine, and arginine) and high levels of serotonin were present in CRPS patients [75]; elevated pro-excitatory amino acids such as L-Aspartate, L-glutamate, L-ornithine [15]; and vascular endothelial growth factor [57] were demonstrated in CRPS patients. Likewise, patients with CRPS also expressed increased levels of pro-excitatory cerebrospinal (CSF) calcium and glutamate [16] (Table 2). The presence of low activity of angiotensin-converting enzyme [51] and low tryptophan was found in CRPS patients [62]. In a single study, patients with CRPS expressed high levels of osteoprotegerin, a glycoprotein central to bone turnover [52].

### 3.9. Risk of Bias Assessment of the Studies

Eleven (19%) of the studies were cross-sectional (Table 3), while most studies were case-control (47/81%) (Table 4). No studies were classified as unsatisfactory based on the Newcastle–Ottawa Scale assessment. The majority were of good quality, with 87.2% (*n* = 41) of case-control studies and 54.5% (*n* = 6) of cross-sectional studies scoring ≥7. The main quality issues identified were the lack of satisfactory justifications and inappropriate selection of cases and controls.

## 4. Discussion

The diagnosis of CRPS is supported by relatively subjective clinical criteria, with no single confirmatory test, which may lead to uncertainty and delays in the diagnosis. In addition, CRPS is heterogeneous with frequent discrepancies between clinical complaints and findings in the physical exam [79]. The most frequently studied biomarkers were related to systemic [29,56,60,62], local [23,35,57,61,69,76], and CNS inflammation [16,17].

As in other predominantly localized inflammation conditions such as organ-specific auto-immune diseases [80], in CRPS, the blood-based general distribution of immune or inflammatory cells [46,60,61,64,65,72,77], and of non-specific inflammatory proteins such as C reactive protein, sedimentation velocity was comparable to controls [64,65,72,77].

On the other hand, our systematic review suggests the existence of a distinct specific immune cellular signature, namely elevated proinflammatory CD14+ CD16+ monocytes [61], reduction in cytotoxic CD8+ lymphocytes and IL-2-producing T cells [46], and of central memory CD8+, CD4+ T lymphocytes [63] has been shown in CRPS. The documentation of elevated biomarkers of pathogenic T-cell activation, such as serum soluble interleukin-2 receptor (sIL-2R) [11,21,64,65], dendritic cell tissue trafficking as p38 phosphorylation [63] further validates the role of specific cell activation in CRPS.

These findings suggest that immune profiling may serve as a valuable tool for improving the diagnosis of CRPS by identifying specific immune cell patterns. Moreover, they open avenues for targeted immunomodulatory therapies that could address the underlying immune dysfunction in CRPS patients.

The results from studies aiming to evaluate the systemic levels of pro-inflammatory cytokines, mostly TNF-α, IL-6, and IL-8, show contradictory results, with some showing higher levels [29,65] and other normal levels [57,58,61,64,72]. The intra- and inter-variation of systemic levels of these pro-inflammatory biomarkers may be caused by inconsistent systemic spill of local inflammatory products [64]. This variation can impact the results in small studies. However, in the CSF the levels of IL-6, IL-1, TNF-α were found to be elevated [16,17,72] in CRPS. The finding of increased pro-inflammatory cytokines, such as TNF-α, IL-6, and IL-1β, in local tissues [76] indicates an ongoing inflammatory response in CRPS. The local infiltration of immune cells [23,34,35,40,69] further reinforces the presence of local inflammation.

The presence of biomarkers of mast cell activation, infiltration [23,34], and elevation of tryptase [23,41] in some, but not all, [73] noted the CRPS-affected tissues can also explain the disproportionate pain in CRPS patients. These biomarkers are not stable throughout the duration of the disease, with the disappearance of mast cell infiltration with chronicity [34]. No differences between CRPS and controls were found for biomarkers such as vasoactive mediators (prostaglandin E2, endothelin-1) [32,41].

Indeed, our systematic review further validates the concept of the dynamic nature of diagnostic inflammatory biomarkers in CRPS [12]. In long-lasting CRPS, the local and systemic levels of proinflammatory cytokines (TNF, IL6, IL-8) decreased with time, with dissipation or absence of the differences to controls in the intermediate to chronic stages [56,76]. The levels of proinflammatory biomarkers are similar among different severity degrees of chronic CRPS [81] and treatment with prednisone caused a reduction in the level of the pro-inflammatory biomarker TNF-α and increasing of the anti-inflammatory IL-10 [82].

The contribution of inflammation is also demonstrated by the documentation of biomarkers of upregulation of mRNA expression levels related to pro-inflammatory cytokines, neuropeptides [57,72], expression of specific pro-inflammatory genes (MMP9, PTGS2, CXCL8, OSM, TLN1) [9,43]. Epigenetic modifications, such as altered DNA methylation of specific genes (*COL11A1*, *HLA-DRB6*) [26] may also be relevant in CRPS.

Of note, administration of infliximab, a TNF-alpha inhibitor in two patients with CRPS was associated with significant improvement in two patients. In both patients, it was documented a parallel local decline of TNF-alpha and IL-6 [83], suggesting that both interleukins can be biomarkers of clinical response. However, in a subsequent clinical trial, the potential of rituximab as a treatment option for CRPS was not confirmed [84].

One can consider that inflammatory biomarkers, particularly in local tissues or CSF, could aid in early diagnosis and disease monitoring in CRPS. Additionally, the dynamic nature of these biomarkers over time supports the need for stage-specific diagnostic and therapeutic approaches, potentially guiding personalized anti-inflammatory approaches.

The levels of calcitonin gene-related peptide, bradykinin, and substance P, but not neurokinin, are consistent with ongoing peripheral nervous system inflammation [22,25,64,65]. CRPS can result from the activation and sensitization of peripheral primary afferents by a local pro-inflammatory environment. One can speculate that in susceptible patients, the initial inflammatory response is not suppressed or is amplified. Neuropeptides further sensitize primary peripheral afferent neurons as well, ascending second-order neurons in the spinal cord [22]. Peripheral sensitization of nociceptors [85], CNS inflammation, and functional adaptations may account for the persistence of hypersensitivity and pain at rest. Accordingly, quantitative sensory tests demonstrate the presence of decreased pain thresholds in the affected side by CRPS [86]. As for interleukins, the levels of neuropeptides change with chronification, with studies showing normalization of CGRP associated with improvement of local inflammation [22], or absence of differences between CRPS and controls, or even lower levels in patients with chronic CRPS [64,65]. For substance P, the shift was also demonstrated with increased levels in acute [64,65] and normal levels in chronic patients [64].

Altered neuropeptide levels may help identify individuals at increased risk of developing CRPS, particularly during pauci-symptomatic or very early stages of the disease.

There are different sequential stages of CRPS, with stage I (first 3 months) characterized by predominant inflammation; stage II (3–6 months); and late-stage or stage III (after 6 months) being characterized by trophic changes [79]. The normalization or decrease in pro-inflammatory biomarkers is in alignment with CRPS chronicity.

Few studies have addressed the possible role of imaging biomarkers in CRPS. Increased FDG uptake in the brain suggests heightened neuroinflammatory activity in pain-processing regions, such as the thalamus and insular cortex [67], and the evidence of local brain functioning or dysfunctional connectivity in the brain regions responsible for motor or sensory processing such as the anterior cingulate cortex, prefrontal cortex, thalamus and amygdala [27,28,37,38,39,48,53,55,68,70] suggest a secondary chronic process of brain maladaptive plasticity in CRPS. The demonstration of a hyperactivated endocannabinoid system with origin in the periphery [47] at least in part, may justify the abnormalities found in CNS, particularly the areas involved in sensory processing.

Although neuroimaging biomarkers primarily reflect secondary phenomena in CRPS patients, they not only enhance our understanding of CRPS pathophysiology but may also serve as valuable tools for assessing clinical response to treatment in chronic cases.

The possible role of autoimmunity is yet to be elucidated. The meaning and potential role as biomarkers of autoantibodies such as IgG anti-myenteric plexus, IgG to SH-SY5Y (inducible autonomic nervous system autoantigen), IgG to P29ING4, IgG to b2 adrenergic and/or the muscarinic-2 receptors, antineuronal IgG, and antinuclear IgG [19,24,30,49,50] need to be clarified. Clarifying the role of these autoantibodies may lead to the development of novel diagnostic biomarkers or CRPS serotypes, potentially improving patient stratification for immunotherapy, which is in line with the current concept of etiological heterogeneity of the disease.

Our review shows that the integration of the different inflammatory, endothelial, epigenetic, and brain potential diagnostic biomarkers in CRPS is complex as they most likely represent different disease mechanisms not necessarily occurring at the same stage of phase CRPS.

## 5. Conclusions

Our review shows that the integration of the different inflammatory, endothelial, epigenetic, and brain potential diagnostic biomarkers in CRPS is complex, as they most likely represent different disease mechanisms, not necessarily occurring at the same stage of CRPS.

## Figures and Tables

**Table 1:** List of inflammation and immune-related potential biomarkers associated with complex regional pain syndrome.

Author, Year	*n*	Cytocines	Cells	Autoantibodies	Neuropeptides	Others
Hartmannsberger et al., 2024 [34]	25		↑Local mast cells and Langerhans cells (acute phase) ±Local mast cells and Langerhans cells (chronic phase)			
Parkitny et al., 2022 [58]	69	±immediate post fracture levels of IL **	±immediate post fracture levels of T Cells			
Bharwani et al., 2020 [21]	23					↑ sIL-2R
Russo et al., 2020 [62]	44	↓ IL-37, ↓ GM-CSF				
Baerlecken et al., 2019 [19]	36			IgG to P29ING4		
Russo et al., 2019 [63]	14		↓ number of central memory CD8+, CD4+ T lymphocytes			↑ p38 signaling in CD1+ mDCs (dendritic cell type activation?)
Bharwani et al., 2017 [11]	80					↑ sIL-2R
Yetişgin et al.,2016 [77]	21		±blood cellular counts			±: VS, CRP
Dirckx et al., 2015 [29]	66	↑ IL-6, TNF-a				
Dirckx et al., 2015 [30]	296			Antineuronal IgG		
				Antinuclear IgG		
Birklein et al., 2014 [23]	55	↑ local IL-6	↑ Local mast cells			↑ local tryptase
		↑ local TNF-α				
Ritz et al., 2011 [61]	25	±proinflammatory cytokines (IL-6, IL-8, TNF-a)	↑ CD14+ CD16+ monocytes			
		±IL-10	±T helper cells (CD4+ CD8−), T cytotoxic cells (CD4− CD8+), NK cells (CD56+), B cells (CD19+), monocytes/macrophages (CD14+)			
Orlova et al., 2011 [57]	41	↑ interleukin1 receptor antagonist				
		↑ monocyte chemotactic protein-1				
		±IL-6, TNFα				
		±Interferon-gamma, IL-1β, IL-2, IL-4, IL-5, IL-7, IL-8, IL-10				
Kohr et al., 2011 [49]	20			IgG to b2 adrenergic and/or the muscarinic-2 receptors		
Kaufmann et al., 2009 [47]	10					↑ anandamide
Kohr et al., 2009 [50]	30			IgG to SH-SY5Y (inducible autonomic nervous system autoantigen)		
Schinkel et al., 2009 [64]	25	± IL-4, IL-6, IL-8, IL-10, IL-11, IL-12	±White Blood Cell Count		↑ Calcitonin Gene-Related Peptide	↑ Soluble TNF Receptor I and II
		± TNF, IL6			↑ Substance P	±CRP
Wesseldijk et al., 2009 [73]	66					±IgE, tryptase
Wesseldijk et al., 2008 [76]	12	↑ local TNF-α				
		↑ local IL-6				
Chronic phase		±IL6, TNF-α				
Kaufmann et al., 2007 [46]	15		±Lymphocites			
			↓ cytotoxic CD8+ lymphocytes; IL-2-producing T cell			
Uçeyler et al., 2007 [72]	40	↓ IL-10, Transforming growth factor beta 1	±Whole blood counts			±CPR
		↑IL-2				
		±TNF-α, IL-6				
		±IL-4				
Alexander et al., 2007 [16]	22	↑ CSF IL-6				
		↓ CSF IL-2, IL-10				
		↑ CSF Monocyte chemoattractant protein-1				
Heijmans-Antonissen et al., 2006 [35]	22	↑ local IL-6				
		↑ local TNF-α				
		±local IFNγ, IL-2, IL-2R, IL-4, IL-5, and IL-10				
		↓eotaxin				
Schinkel et al., 2006 [65]	25	↑ IL-8	±leukocytes		↑ Substance P	↓ soluble forms of selectins
		±IL-6			±Neuropeptide Y	±CRP
					±CGRP	↑ soluble tumor necrosis factor receptor I/II
Tan et al., 2005 [69]	6		↑ Local leukocytes			
Alexander et al., 2005 [17]	24	↑ CSF IL-6/IL-1				
		±CSF TNF-α				
Munnikes et al., 2005 [56]	25	↑ local IL-6				
		↑ local TNF-α				
Chronic phase		±local IL-6				
Chronic phase		±local TNF-α				
Blaes et al., 2004 [24]	12			↑ IgG Myenteric plexus		
Huygen et al., 2004 [41]	20	↑ local IL-6				↑ tryptase
		↑ local TNF-α				
Huygen et al., 2002 [40]	9	↑ local IL-6				
		↑ local TNF-α				
		±local IL-1b, IL-1b				
Birklein et al., 2001 [22]	19				↑ Calcitonin Gene-Related Peptide	
Ribbers et al., 1998 [60]	13		±Cell distribution (B and T lymphocyte populations)			
Blair et al., 1998 [25]	61				↑ Calcitonin Gene-Related Peptide	
					±Neurokinin	
					↑Bradykinin	

↑: increased; ↓: decreased; ±: no significant difference; CSF: Cerebrospinal fluid; IL: Interleukins; **: IL-1β, IL-10, IFN-α, IL-6, IL-12, RANTES, IL-13, IL-15, IL-17, MIP-1α, GM-CSF, MIP-1β; MCP-1, IL-5, IFN-γ, TNF-α, IL-1Ra, IL-2 , IL-7, IP-10 , IL-2r, MIG , IL-4  =  interleukin-4, IL-8; GM-CSF: Granulocyte-macrophage colony-stimulating factor; TNF: Tumor necrosis factor; ± No difference/correlation; VS: erythrocyte sedimentation rate; CRP: C-reactive protein levels.

**Table 2:** Genetic, epigenetic, brain imaging, and functional neurophysiological biomarkers associated with complex regional pain syndrome.

Author, Year	*n*	Genetic and Epigenetics	Brain Imaging		Functional Neurophysiological	Other Biomarkers
			Structural	Metabolic		
Hok et al., 2024 [36]	51				↓ antinociceptive modulation via the brainstem antinociceptive system	
Shaikh et al., 2024 [66]		Single-nucleotide polymorphism of genes ANO10, P2RX7, PRKAG1 and SLC12A9				
Hotta et al., 2023 [39]	17				Sustained somatotopic alteration of the somatosensory cortex	
Delon-Martin et al., 2023 [27]	11				↑ localized activation in the primary somatosensory cortex (transcranial magnetic stimulation)	
Zhu et al., 2023 [9]	9	Five top five hub genes: MMP9, PTGS2, CXCL8, OSM, TLN1				
Hong et al., 2023 [37]	21				↑ functional connectivity in the somatosensory (S1) subnetworks	
					↓functional connectivity in the prefronto-parieto-cingulo-thalamic subnetworks	
Lee et al., 2022 [53]	15				↑ Basal ganglia infra-slow oscillations	
					↑ Basal ganglia resting connectivity	
Domin et al., 2021 [31]	24				↓ insula and bilateral grey matter medial thalamus.	
König et al., 2021 [51]	25					↓ activity of angiotensin-converting enzyme
Azqueta-Gavaldon et al., 2020 [18]	20				↓gray matter density in the putamen/functional connectivity increases amongst the putamen and pre-/postcentral gyri and cerebellum	
Russo et al., 2020 [62]	44					↓ tryptophan
Di Pietro et al., 2020 [28]	15				↑ thalamo-S1 functional connectivity	
Bruehl et al., 2019 [26]	9	Altered methylation of specific genes (COL11A1 and HLA-DRB6)				
Jung et al., 2019 [44]	12			Disruption of interactions between specific central and metabolic metabolites * in the thalamus		
Kohle et al., 2019 [48]	15				↓ activation of subthalamic nucleus, nucleus accumbens, and putamen	
Jung, et al., 2018 [45]	12			Anormal interactions of lipid13a and L f lipid 09 in the thalamus with peripheral tCr		
Hotta et al., 2017 [38]	13				Abnormal neural activity in sensorimotor and pain-related areas	
Shokouhi et al., 2017 [68]	28		↓grey matter in somatosensory cortex, and limbic system	↓ perfusion in somatosensory cortex, and limbic system (early phase)		
				↑ perfusion in somatosensory cortex, and limbic system (late phase)		
Janicki et al., 2016 [42]	230	±Common Single Nucleotide Polymorphisms				
Zhou et al., 2015 [78]	35		↑ volume of choroid plexus			
Lee et al., 2015 [54]	25		↓ cortical thinning in the prefrontal cortex			
Pleger et al., 2014 [59]	15		↑ in gray matter density in dorsomedial prefrontal			
			↑ in gray matter density located in the primary motor cortex (contralateral to the affected limb)			
Krämer et al., 2014 [52]	33					↑ Osteoprotegerin
Barad et al., 2013 [20]	15				↓ Grey matter volume in pain-related areas (dorsal insula, orbitofrontal cortex, cingulate cortex.	
Jin et al., 2013 [43]	24	Increased expression of MMP9				
Alexander et al., 2013 [15]	160					↑ AA: L-Aspartate, L-glutamate, L-ornithine
						↓ L-tryptophan and L-arginine
Lenz et al., 2011 [55]	21				↓ Somatosensory cortex inhibition	
Orlova et al., 2011 [57]	41	↑ Specific microRNA: hsa-miR-532-3p				↑ Vascular endothelial growth factor
Walton et al., 2010 [71]	64				Altered magneto-encephalographic imaging (thalamo-cortical Dysrhythmia)	
Wesseldijk et al., 2008 [75]	64					↑ NMDA excitatory amino acids: glutamate, glutamine, glycine, taurine, and arginine
Wesseldijk et al., 2008 [74]	35					↑ serotonin
Geha et al., 2008 [33]	26				↓ insula, ventromedial prefrontal cortex, nucleus accumbens; fractional anisotropy in cingulum-callosal bundle	
Turton et al., 2007 [70]	8				↓ motor response to TMS	
Alexander et al., 2007 [16]	22					↑ CSF Calcium and glutamate
						↑ CSF Glial fibrillary acidic protein
						↑ CSF Nitric oxide metabolites
Uçeyler et al., 2007 [72]	40	↓ mRNA IL-4, IL-8, IL-10				
		± transforming growth factor-b1mRNA				
		↑ TNF and IL-2 mRNA level				
Janicki et al., 2016 [42]	230	±Common Single Nucleotide Polymorphisms				
Shiraishi et al., 2006 [67]	18			↑ activity in somatosensory cortex		
				↓ contralateral activity in specific motor areas		
Huygen et al., 2004 [41]	20					±prostaglandin E2
Eisenberg et al., 2004 [32]	38					±Endothelin-1

↑: increased; ↓: decreased; ±: no significant difference; CSF: Cerebrospinal fluid; IL: Interleukins; tCr = total creatine levels; * N-acetylaspartate, tCr, and potassium; TMS: transmagnetic stimulation; AA: Amino acids; ± No difference/correlation.

**Table 3:** Risk of bias assessment of cross-sectional studies based on the Newcastle–Ottawa scale.

	Newcastle–Ottawa Scale Items						
Study	S1	S2	S3	S4	C	O	Total
Hartmannsberger et al., 2024 [34]	*	-	*	**	*	**	7
Delon-Martin et al., 2024 [27]	*	-	*	**	*	**	7
Bharwani et al., 2020 [21]	*	-	*	**	*	**	7
Baerlecken et al., 2019 [19]	*	-	*	**	*	***	9
Dirckx et al., 2015 [30]	*	-	*	**	*	**	7
Kohr et al., 2011 [49]	*	-	*	**	*	*	6
Alexander et al., 2007 [16]	*	-	*	**	-	*	5
Heijmans-Antonissen et al., 2006 [35]	*	-	*	**	*	*	6
Alexander et al., 2005 [17]	*	-	*	**	*	**	7
Blaes et al., 2004 [24]	*	-	*	*	*	*	5
Blair et al., 1998 [25]	*	-	*	*	*	*	5

* resemble each point; Abbreviations: S = Selection; S1, representativeness; S2, selection of the unexposed; S3, exposure determination; S4, outcome not present at the beginning of the study C: Comparability; B; O: Outcomes.

**Table 4:** Risk of bias assessment of case controls based on the Newcastle–Ottawa scale.

	Newcastle–Ottawa Scale Items						
Study	S1	S2	S3	S4	C	E	Total
Shaikh et al., 2024 [66]	*	*	*	*	**	***	9
Hok et al., 2024 [36]	*	*	-	*	**	**	7
Hotta et al., 2023 [39]	*	*	*	*	**	**	8
Hong et al., 2023 [37]	*	*	*	*	**	**	8
Zhu et al., 2023 [9]	*	*	*	*	**	***	9
Lee et al., 2022 [53]	*	*	*	*	**	**	8
Parkitny et al., 2022 [58]	*	*	-	*	**	***	8
Orlova et al., 2011 [57]	*	*	-	*	**	**	7
König et al., 2021 [51]	*	*	-	*	**	**	7
Domin et al., 2021 [31]							
Azqueta-Gavaldon et al., 2020 [18]	*	*	-	*	**	**	7
Russo et al., 2020 [62]	*	*	-	*	**	**	7
Di Pietro et al., 2020 [28]	*	*	-	*	**	**	7
Russo et al., 2019 [63]	*	*	-	*	*	**	6
Kohler et al., 2019 [48]	*	*	*	*	**	**	8
Jung et al., 2019 [44]	*	*	-	*	**	**	7
Jung et al., 2018 [45]	*	*	-	*	*	**	6
Bruehl et al., 2019 [26]	*	*	-	*	**	***	8
Wesseldijk et al., 2009 [73]	*	*	*	*	**	***	9
Wesseldijk et al., 2008 [76]	*	*	-	*	**	**	7
Shokouhi et al., 2017 [68]	*	*	-	*	**	**	7
Bharwani et al., 2017 [11]	*	*	-	*	**	**	7
Hotta et al., 2017 [38]	*	*	-	*	**	*	6
Yetişgin et al., 2016 [77]	*	*	-	*	**	**	7
Zhou et al., 2015 [78]	*	*	-	*	**	**	7
Lee et al., 2015 [54]	*	*	*	*	**	**	8
Dirckx et al., 2015 [29]	*	*	-	*	**	**	7
Barad et al., 2014 [20]	*	*	-	*	**	**	7
Krämer et al., 2014 [52]	*	*	-	*	**	**	7
Birklein et al., 2014 [23]	*	*	*	*	**	**	8
Pleger et al., 2014 [59]	*	*	*	*	**	**	8
Jin et al., 2013 [43]	*	*	*	*	**	***	9
Alexander et al., 2013 [15]	*	-	-	*	**	**	6
Lenz et al., 2011 [55]	*	*	*	*	**	***	9
Ritz et al., 2011 [61]	*	*	*	*	**	**	8
Walton et al., 2010 [71]	*	*	-	*	**	**	7
Kaufmann et al., 2009 [47]	*	-	-	*	*	**	5
Kohr et al., 2009 [50]	*	*	*	*	**	**	8
Schinkel et al., 2009 [64]	*	*	*	*	**	**	8
Geha et al., 2008 [33]	*	*	-	*	**	**	7
Wesseldijk et al., 2008 [75]	*	*	-	*	**	**	7
Wesseldijk et al., 2008 b [74]	*	*	-	*	**	**	7
Kaufmann et al.,2007 [46]	*	-	-	*	*	***	6
Uçeyler et al., 2007 [72]	*	*	-	*	**	**	7
Turton et al., 2007 [70]	*	*	-	*	**	**	7
Janicki et al., 2016 [42]	*	*	-	*	**	**	7
Schinkel et al., 2006 [65]	*	*	*	*	**	***	9
Shiraishi et al., 2006 [67]	*	*	-	*	**	**	7
Munnikes et al., 2005 [56]	*	-	-	*	**	**	6
Tan et al., 2005 [69]	*	*	-	*	**	**	7
Eisenberg et al., 2004 [32]	*	*	-	*	**	***	8
Huygen et al., 2004 [41]	*	*	-	*	**	**	7
Huygen et al., 2002 [40]	*	*	-	*	**	**	7
Birklein et al., 2001 [22]	*	*	-	*	**	**	7
Ribbers et al., 1998 [60]	*	*	*	*	**	**	8

* resemble each point; Abbreviations: S1 case definition; S2 case representativeness; S3 control selection, S4 control definition; C: Comparability; E, Exposure. Green ≥7 (good); Orange: 5.6 (satisfactory); red: ≤4 (unsatisfactory).

## References

[1] Van Velzen G.A.J., Perez R.S.G.M., Van Gestel M.A., Huygen F.J.P.M., Van Kleef M., Van Eijs F., Dahan A., Van Hilten J.J., Marinus J. (2014). Health-Related Quality of Life in 975 Patients with Complex Regional Pain Syndrome Type 1. Pain.

[2] D’Souza R.S., Klasova J., Saini C., Chang A., Music S., Shah J.D., Elmati P.R., Chitneni A., To J., Prokop L.J. (2025). Global Burden of Complex Regional Pain Syndrome in At-Risk Populations: Estimates of Prevalence From 35 Countries Between 1993 and 2023. Anesth. Analg..

[3] Harden R.N., McCabe C.S., Goebel A., Massey M., Suvar T., Grieve S., Bruehl S. (2022). Complex Regional Pain Syndrome: Practical Diagnostic and Treatment Guidelines, 5th Edition. Pain Med..

[4] Goebel A., Barker C., Birklein F., Brunner F., Casale R., Eccleston C., Eisenberg E., McCabe C.S., Moseley G.L., Perez R. (2019). Standards for the Diagnosis and Management of Complex Regional Pain Syndrome: Results of a European Pain Federation Task Force. Eur. J. Pain.

[5] Limerick G., Christo D.K., Tram J., Moheimani R., Manor J., Chakravarthy K., Karri J., Christo P.J. (2023). Complex Regional Pain Syndrome: Evidence-Based Advances in Concepts and Treatments. Curr. Pain Headache Rep..

[6] Ghaly L., Bargnes V., Rahman S., Tawfik G.-A., Bergese S., Caldwell W. (2023). Interventional Treatment of Complex Regional Pain Syndrome. Biomedicines.

[7] Bruehl S. (2015). Complex Regional Pain Syndrome. BMJ.

[8] Mangnus T.J.P., Bharwani K.D., Dirckx M., Huygen F.J.P.M. (2022). From a Symptom-Based to a Mechanism-Based Pharmacotherapeutic Treatment in Complex Regional Pain Syndrome. Drugs.

[9] Zhu H., Wen B., Xu L., Huang Y. (2023). Identification of Potential Inflammation-Related Genes and Key Pathways Associated with Complex Regional Pain Syndrome. Biomolecules.

[10] Ma T., Li Z.-Y., Yu Y., Yang Y., Ni M.-H., Xie H., Wang W., Huang Y.-X., Li J.-L., Cui G.-B. (2022). Gray Matter Abnormalities in Patients with Complex Regional Pain Syndrome: A Systematic Review and Meta-Analysis of Voxel-Based Morphometry Studies. Brain Sci..

[11] Bharwani K.D., Dirckx M., Stronks D.L., Dik W.A., Schreurs M.W.J., Huygen F.J.P.M. (2017). Elevated Plasma Levels of sIL-2R in Complex Regional Pain Syndrome: A Pathogenic Role for T-Lymphocytes?. Mediat. Inflamm..

[12] Parkitny L., McAuley J.H., Di Pietro F., Stanton T.R., O’Connell N.E., Marinus J., Van Hilten J.J., Moseley G.L. (2013). Inflammation in Complex Regional Pain Syndrome: A Systematic Review and Meta-Analysis. Neurology.

[13] Birklein F., Schmelz M. (2008). Neuropeptides, Neurogenic Inflammation and Complex Regional Pain Syndrome (CRPS). Neurosci. Lett..

[14] Well G., Shea B., O’Connell D., Peterson J., Welch V., Losos M., Tugwell P. The NewcastleOttawa Scale (NOS) for Assessing the Quality of Nonrandomized Studies in Meta-Analysis. https://www.ohri.ca/programs/clinical_epidemiology/oxford.asp.

[15] Alexander G.M., Reichenberger E., Peterlin B.L., Perreault M.J., Grothusen J.R., Schwartzman R.J. (2013). Plasma Amino Acids Changes in Complex Regional Pain Syndrome. Pain Res. Treat..

[16] Alexander G.M., Perreault M.J., Reichenberger E.R., Schwartzman R.J. (2007). Changes in Immune and Glial Markers in the CSF of Patients with Complex Regional Pain Syndrome. Brain. Behav. Immun..

[17] Alexander G.M., van Rijn M.A., van Hilten J.J., Perreault M.J., Schwartzman R.J. (2005). Changes in Cerebrospinal Fluid Levels of Pro-Inflammatory Cytokines in CRPS. Pain.

[18] Azqueta-Gavaldon M., Youssef A.M., Storz C., Lemme J., Schulte-Göcking H., Becerra L., Azad S.C., Reiners A., Ertl-Wagner B., Borsook D. (2020). Implications of the Putamen in Pain and Motor Deficits in Complex Regional Pain Syndrome. Pain.

[19] Baerlecken N.T., Gaulke R., Pursche N., Witte T., Karst M., Bernateck M. (2019). Autoantibodies against P29ING4 Are Associated with Complex Regional Pain Syndrome. Immunol. Res..

[20] Barad M.J., Ueno T., Younger J., Chatterjee N., Mackey S. (2014). Complex Regional Pain Syndrome Is Associated with Structural Abnormalities in Pain-Related Regions of the Human Brain. J. Pain.

[21] Bharwani K.D., Dirckx M., Stronks D.L., Dik W.A., Huygen F.J.P.M., Dozio E. (2020). Serum Soluble Interleukin-2 Receptor Does Not Differentiate Complex Regional Pain Syndrome from Other Pain Conditions in a Tertiary Referral Setting. Mediat. Inflamm..

[22] Birklein F., Schmelz M., Schifter S., Weber M. (2001). The Important Role of Neuropeptides in Complex Regional Pain Syndrome. Neurology.

[23] Birklein F., Drummond P.D., Li W., Schlereth T., Albrecht N., Finch P.M., Dawson L.F., Clark J.D., Kingery W.S. (2014). Activation of Cutaneous Immune Responses in Complex Regional Pain Syndrome. J. Pain.

[24] Blaes F., Schmitz K., Tschernatsch M., Kaps M., Krasenbrink I., Hempelmann G., Bräu M.E. (2004). Autoimmune Etiology of Complex Regional Pain Syndrome (M. Sudeck). Neurology.

[25] Blair S.J., Chinthagada M., Hoppenstehdt D., Kijowski R., Fareed J. (1998). Role of Neuropeptides in Pathogenesis of Reflex Sympathetic Dystrophy. Acta Orthop. Belg..

[26] Bruehl S., Gamazon E.R., Van De Ven T., Buchheit T., Walsh C.G., Mishra P., Ramanujan K., Shaw A. (2019). DNA Methylation Profiles Are Associated with Complex Regional Pain Syndrome after Traumatic Injury. Pain.

[27] Delon-Martin C., Lefaucheur J.-P., Hodaj E., Sorel M., Dumolard A., Payen J.-F., Hodaj H. (2024). Neural Correlates of Pain-Autonomic Coupling in Patients With Complex Regional Pain Syndrome Treated by Repetitive Transcranial Magnetic Stimulation of the Motor Cortex. Neuromodul. J. Int. Neuromodul. Soc..

[28] Di Pietro F., Lee B., Henderson L.A. (2020). Altered Resting Activity Patterns and Connectivity in Individuals with Complex Regional Pain Syndrome. Hum. Brain Mapp..

[29] Dirckx M., Stronks D.L., van Bodegraven-Hof E.a.M., Wesseldijk F., Groeneweg J.G., Huygen F.J.P.M. (2015). Inflammation in Cold Complex Regional Pain Syndrome. Acta Anaesthesiol. Scand..

[30] Dirckx M., Schreurs M.W.J., de Mos M., Stronks D.L., Huygen F.J.P.M. (2015). The Prevalence of Autoantibodies in Complex Regional Pain Syndrome Type I. Mediators Inflamm..

[31] Domin M., Strauss S., McAuley J.H., Lotze M. (2021). Complex Regional Pain Syndrome: Thalamic GMV Atrophy and Associations of Lower GMV With Clinical and Sensorimotor Performance Data. Front. Neurol..

[32] Eisenberg E., Erlich T., Zinder O., Lichinsky S., Diamond E., Pud D., Davar G. (2004). Plasma Endothelin-1 Levels in Patients with Complex Regional Pain Syndrome. Eur. J. Pain Lond. Engl..

[33] Geha P.Y., Baliki M.N., Harden R.N., Bauer W.R., Parrish T.B., Apkarian A.V. (2008). The Brain in Chronic CRPS Pain: Abnormal Gray-White Matter Interactions in Emotional and Autonomic Regions. Neuron.

[34] Hartmannsberger B., Scriba S., Guidolin C., Becker J., Mehling K., Doppler K., Sommer C., Rittner H.L. (2024). Transient Immune Activation without Loss of Intraepidermal Innervation and Associated Schwann Cells in Patients with Complex Regional Pain Syndrome. J. Neuroinflamm..

[35] Heijmans-Antonissen C., Wesseldijk F., Munnikes R.J., Huygen F.J., van der Meijden P., Hop W.C.J., Hooijkaas H., Zijlstra F.J. (2006). Multiplex Bead Array Assay for Detection of 25 Soluble Cytokines in Blister Fluid of Patients with Complex Regional Pain Syndrome Type 1. Mediators Inflamm..

[36] Hok P., Strauss S., McAuley J., Domin M., Wang A.P., Rae C., Moseley G.L., Lotze M. (2024). Functional Connectivity in Complex Regional Pain Syndrome: A Bicentric Study. NeuroImage.

[37] Hong H., Suh C., Namgung E., Ha E., Lee S., Kim R.Y., Song Y., Oh S., Lyoo I.K., Jeong H. (2023). Aberrant Resting-State Functional Connectivity in Complex Regional Pain Syndrome: A Network-Based Statistics Analysis. Exp. Neurobiol..

[38] Hotta J., Saari J., Koskinen M., Hlushchuk Y., Forss N., Hari R. (2017). Abnormal Brain Responses to Action Observation in Complex Regional Pain Syndrome. J. Pain.

[39] Hotta J., Saari J., Harno H., Kalso E., Forss N., Hari R. (2023). Somatotopic Disruption of the Functional Connectivity of the Primary Sensorimotor Cortex in Complex Regional Pain Syndrome Type 1. Hum. Brain Mapp..

[40] Huygen F.J.P.M., De Bruijn A.G.J., De Bruin M.T., Groeneweg J.G., Klein J., Zijlstra F.J. (2002). Evidence for Local Inflammation in Complex Regional Pain Syndrome Type 1. Mediat. Inflamm..

[41] Huygen F.J.P.M., Ramdhani N., van Toorenenbergen A., Klein J., Zijlstra F.J. (2004). Mast Cells Are Involved in Inflammatory Reactions during Complex Regional Pain Syndrome Type 1. Immunol. Lett..

[42] Janicki P.K., Alexander G.M., Eckert J., Postula M., Schwartzman R.J. (2016). Analysis of Common Single Nucleotide Polymorphisms in Complex Regional Pain Syndrome: Genome Wide Association Study Approach and Pooled DNA Strategy. Pain Med..

[43] Jin E.-H., Zhang E., Ko Y., Sim W.S., Moon D.E., Yoon K.J., Hong J.H., Lee W.H. (2013). Genome-Wide Expression Profiling of Complex Regional Pain Syndrome. PLoS ONE.

[44] Jung Y.-H., Kim H., Lee D., Lee J.-Y., Lee W.J., Moon J.Y., Kim Y.C., Choi S.-H., Kang D.-H. (2019). Disruption of Homeostasis Based on the Right and Left Hemisphere in Patients with Complex Regional Pain Syndrome. Neuroimmunomodulation.

[45] Jung Y.-H., Kim H., Jeon S.Y., Kwon J.M., Lee D., Choi S.-H., Kang D.-H. (2018). Aberrant Interactions of Peripheral Measures and Neurometabolites with Lipids in Complex Regional Pain Syndrome Using Magnetic Resonance Spectroscopy: A Pilot Study. Mol. Pain.

[46] Kaufmann I., Eisner C., Richter P., Huge V., Beyer A., Chouker A., Schelling G., Thiel M. (2007). Lymphocyte Subsets and the Role of Th1/Th2 Balance in Stressed Chronic Pain Patients. Neuroimmunomodulation.

[47] Kaufmann I., Hauer D., Huge V., Vogeser M., Campolongo P., Chouker A., Thiel M., Schelling G. (2009). Enhanced Anandamide Plasma Levels in Patients with Complex Regional Pain Syndrome Following Traumatic Injury: A Preliminary Report. Eur. Surg. Res. Eur. Chir. Forsch. Rech. Chir. Eur..

[48] Kohler M., Strauss S., Horn U., Langner I., Usichenko T., Neumann N., Lotze M. (2019). Differences in Neuronal Representation of Mental Rotation in Patients With Complex Regional Pain Syndrome and Healthy Controls. J. Pain.

[49] Kohr D., Singh P., Tschernatsch M., Kaps M., Pouokam E., Diener M., Kummer W., Birklein F., Vincent A., Goebel A. (2011). Autoimmunity against the Β2 Adrenergic Receptor and Muscarinic-2 Receptor in Complex Regional Pain Syndrome. Pain.

[50] Kohr D., Tschernatsch M., Schmitz K., Singh P., Kaps M., Schäfer K.-H., Diener M., Mathies J., Matz O., Kummer W. (2009). Autoantibodies in Complex Regional Pain Syndrome Bind to a Differentiation-Dependent Neuronal Surface Autoantigen. Pain.

[51] König S., Steinebrey N., Herrnberger M., Escolano-Lozano F., Schlereth T., Rebhorn C., Birklein F. (2021). Reduced Serum Protease Activity in Complex Regional Pain Syndrome: The Impact of Angiotensin-Converting Enzyme and Carboxypeptidases. J. Pharm. Biomed. Anal..

[52] Krämer H.H., Hofbauer L.C., Szalay G., Breimhorst M., Eberle T., Zieschang K., Rauner M., Schlereth T., Schreckenberger M., Birklein F. (2014). Osteoprotegerin: A New Biomarker for Impaired Bone Metabolism in Complex Regional Pain Syndrome?. Pain.

[53] Lee B., Di Pietro F., Henderson L.A., Austin P.J. (2022). Altered Basal Ganglia Infraslow Oscillation and Resting Functional Connectivity in Complex Regional Pain Syndrome. J. Neurosci. Res..

[54] Lee D.-H., Lee K.-J., Cho K.I.K., Noh E.C., Jang J.H., Kim Y.C., Kang D.-H. (2015). Brain Alterations and Neurocognitive Dysfunction in Patients With Complex Regional Pain Syndrome. J. Pain.

[55] Lenz M., Höffken O., Stude P., Lissek S., Schwenkreis P., Reinersmann A., Frettlöh J., Richter H., Tegenthoff M., Maier C. (2011). Bilateral Somatosensory Cortex Disinhibition in Complex Regional Pain Syndrome Type I. Neurology.

[56] Munnikes R.J.M., Muis C., Boersma M., Heijmans-Antonissen C., Zijlstra F.J., Huygen F.J.P.M. (2005). Intermediate Stage Complex Regional Pain Syndrome Type 1 Is Unrelated to Proinflammatory Cytokines. Mediators Inflamm..

[57] Orlova I.A., Alexander G.M., Qureshi R.A., Sacan A., Graziano A., Barrett J.E., Schwartzman R.J., Ajit S.K. (2011). MicroRNA Modulation in Complex Regional Pain Syndrome. J. Transl. Med..

[58] Parkitny L., McAuley J.H., Herbert R.D., Di Pietro F., Cashin A.G., Ferraro M.C., Moseley G.L. (2022). Post-Fracture Serum Cytokine Levels Are Not Associated with a Later Diagnosis of Complex Regional Pain Syndrome: A Case-Control Study Nested in a Prospective Cohort Study. BMC Neurol..

[59] Pleger B., Draganski B., Schwenkreis P., Lenz M., Nicolas V., Maier C., Tegenthoff M. (2014). Complex Regional Pain Syndrome Type I Affects Brain Structure in Prefrontal and Motor Cortex. PLoS ONE.

[60] Ribbers G.M., Oosterhuis W.P., van Limbeek J., de Metz M. (1998). Reflex Sympathetic Dystrophy: Is the Immune System Involved?. Arch. Phys. Med. Rehabil..

[61] Ritz B.W., Alexander G.M., Nogusa S., Perreault M.J., Peterlin B.L., Grothusen J.R., Schwartzman R.J. (2011). Elevated Blood Levels of Inflammatory Monocytes (CD14+ CD16+) in Patients with Complex Regional Pain Syndrome. Clin. Exp. Immunol..

[62] Russo M.A., Georgius P., Pires A.S., Heng B., Allwright M., Guennewig B., Santarelli D.M., Bailey D., Fiore N.T., Tan V.X. (2020). Novel Immune Biomarkers in Complex Regional Pain Syndrome. J. Neuroimmunol..

[63] Russo M.A., Fiore N.T., Van Vreden C., Bailey D., Santarelli D.M., McGuire H.M., Fazekas De St Groth B., Austin P.J. (2019). Expansion and Activation of Distinct Central Memory T Lymphocyte Subsets in Complex Regional Pain Syndrome. J. Neuroinflamm..

[64] Schinkel C., Scherens A., Köller M., Roellecke G., Muhr G., Maier C. (2009). Systemic Inflammatory Mediators in Post-Traumatic Complex Regional Pain Syndrome (CRPS I)—Longitudinal Investigations and Differences to Control Groups. Eur. J. Med. Res..

[65] Schinkel C., Gaertner A., Zaspel J., Zedler S., Faist E., Schuermann M. (2006). Inflammatory Mediators Are Altered in the Acute Phase of Posttraumatic Complex Regional Pain Syndrome. Clin. J. Pain.

[66] Shaikh S.S., Goebel A., Lee M.C., Nahorski M.S., Shenker N., Pamela Y., Drissi I., Brown C., Ison G., Shaikh M.F. (2024). Evidence of a Genetic Background Predisposing to Complex Regional Pain Syndrome Type 1. J. Med. Genet..

[67] Shiraishi S., Kobayashi H., Nihashi T., Kato K., Iwano S., Nishino M., Ishigaki T., Ikeda M., Kato T., Ito K. (2006). Cerebral Glucose Metabolism Change in Patients with Complex Regional Pain Syndrome: A PET Study. Radiat. Med..

[68] Shokouhi M., Clarke C., Morley-Forster P., Moulin D.E., Davis K.D., St Lawrence K. (2018). Structural and Functional Brain Changes at Early and Late Stages of Complex Regional Pain Syndrome. J. Pain.

[69] Tan E.C.T.H., Oyen W.J.G., Goris R.J.A. (2005). Leukocytes in Complex Regional Pain Syndrome Type I. Inflammation.

[70] Turton A.J., McCabe C.S., Harris N., Filipovic S.R. (2007). Sensorimotor Integration in Complex Regional Pain Syndrome: A Transcranial Magnetic Stimulation Study. Pain.

[71] Walton K.D., Dubois M., Llinás R.R. (2010). Abnormal Thalamocortical Activity in Patients with Complex Regional Pain Syndrome (CRPS) Type I. Pain.

[72] Üçeyler N., Eberle T., Rolke R., Birklein F., Sommer C. (2007). Differential Expression Patterns of Cytokines in Complex Regional Pain Syndrome. Pain.

[73] Wesseldijk F., van Toorenenbergen A.W., van Wijk R.G., Huygen F.J., Zijlstra F.J. (2009). IgE-Mediated Hypersensitivity: Patients with Complex Regional Pain Syndrome Type 1 (CRPS1) vs the Dutch Population. A Retrospective Study. Pain Med..

[74] Wesseldijk F., Fekkes D., Huygen F.J., Bogaerts-Taal E., Zijlstra F.J. (2008). Increased Plasma Serotonin in Complex Regional Pain Syndrome Type 1. Anesth. Analg..

[75] Wesseldijk F., Fekkes D., Huygen F.J.P.M., van de Heide-Mulder M., Zijlstra F.J. (2008). Increased Plasma Glutamate, Glycine, and Arginine Levels in Complex Regional Pain Syndrome Type 1. Acta Anaesthesiol. Scand..

[76] Wesseldijk F., Huygen F.J.P.M., Heijmans-Antonissen C., Niehof S.P., Zijlstra F.J. (2008). Six Years Follow-up of the Levels of TNF-Alpha and IL-6 in Patients with Complex Regional Pain Syndrome Type 1. Mediat. Inflamm..

[77] Yetişgin A., Tutoğlu A., Cinakli A., Kul M., Boyaci A. (2016). Platelet and Erythrocyte Indexes in Complex Regional Pain Syndrome Type I. Arch. Rheumatol..

[78] Zhou G., Hotta J., Lehtinen M.K., Forss N., Hari R. (2015). Enlargement of Choroid Plexus in Complex Regional Pain Syndrome. Sci. Rep..

[79] Bruehl S., Harden R.N., Galer B.S., Saltz S., Backonja M., Stanton-Hicks M. (2002). Complex Regional Pain Syndrome: Are There Distinct Subtypes and Sequential Stages of the Syndrome?. Pain.

[80] Lesage S., Goodnow C.C. (2001). Organ-Specific Autoimmune Disease. J. Exp. Med..

[81] Mangnus T.J.P., Bharwani K.D., Dik W.A., Baart S.J., Dirckx M., Huygen F.J.P.M. (2023). Is There an Association between Serum Soluble Interleukin-2 Receptor Levels and Syndrome Severity in Persistent Complex Regional Pain Syndrome?. Pain Med..

[82] Kalita J., Shukla R., Pandey P.C. (2024). Effect of Prednisolone on Clinical and Cytokine mRNA Profiling in Complex Regional Pain Syndrome. J. Mol. Neurosci. MN.

[83] Huygen F.J.P.M., Niehof S., Zijlstra F.J., van Hagen P.M., van Daele P.L.A. (2004). Successful Treatment of CRPS 1 with Anti-TNF. J. Pain Symptom Manag..

[84] Dirckx M., Groeneweg G., Wesseldijk F., Stronks D.L., Huygen F.J.P.M. (2013). Report of a Preliminary Discontinued Double-Blind, Randomized, Placebo-Controlled Trial of the Anti-TNF-α Chimeric Monoclonal Antibody Infliximab in Complex Regional Pain Syndrome. Pain Pract. Off. J. World Inst. Pain.

[85] Orstavik K. (2003). Pathological C-Fibres in Patients with a Chronic Painful Condition. Brain.

[86] Tahmoush A.J., Schwartzman R.J., Hopp J.L., Grothusen J.R. (2000). Quantitative Sensory Studies in Complex Regional Pain Syndrome Type 1/RSD. Clin. J. Pain.

